# Stratified Threshold Values of QuantiFERON Assay for Diagnosing Tuberculosis Infection in Immunocompromised Populations

**DOI:** 10.1155/2011/940642

**Published:** 2011-06-27

**Authors:** Haruyuki Ariga, Hideaki Nagai, Atsuyuki Kurashima, Yoshihiko Hoshino, Syunsuke Shoji, Yutsuki Nakajima

**Affiliations:** ^1^Center for Respiratory Medicine, National Hospital Organization, Tokyo National Hospital, 3-1-1 Takeoka, Kiyose, Tokyo 204-8585, Japan; ^2^Department of Mycobacteriology, National Institute of Infectious Diseases, 4-2-1 Aoba, Higashi-Murayama, Tokyo 189-0002, Japan

## Abstract

*Background*. The detection of latent tuberculosis (TB) is essential for TB control, but T-cell assay might be influenced by degree of immunosuppression. The relationship between immunocompetence and interferon (IFN)-*γ* response in QuantiFERON-TB Gold (QFT) is uncertain, especially in HIV-negative populations. *Methods and Results*. QFT has been performed for healthy subjects and TB suspected patients. Of 3017 patients, 727 were diagnosed as pulmonary TB by culture. The absolute number of blood lymphocyte in TB patients was significantly associated with QFT. Definitive TB patients were divided into eight groups according to lymphocyte counts. For each subgroup, receiver operating characteristic curve analysis was conducted from 357 healthy control subjects. The optimal cut-off for the patient group with adequate lymphocyte counts was found, but this was reduced for lymphocytopenia. *Conclusions*. The lymphocyte count was positively associated with QFT. Positive criteria should be calibrated in consideration of cell-mediated immunocompetence and risk of progression to active TB.

## 1. Introduction

Treatment of persons with latent tuberculosis infection (LTBI) is essential to prevent the development of active tuberculosis (TB). Although the greatest risk of TB disease is traditionally associated with recent exposures, immunocompromised hosts represent a setting in which reactivation of TB from more remote exposures is a concern. The US Centers for Disease Control and Prevention (CDC) recommends targeted testing for LTBI in specific at-risk groups, including immunocompromised individuals with HIV infection, receiving immunosuppressive therapy, chronic renal failure, solid organ transplant, silicosis, cancer of the head or neck and lung, malnutrition, and diabetes [1]. The targeted treatment of LTBI in these populations is also central to TB control at the public health level [1, 2]. 

Until recently, tuberculin skin testing (TST) was the only test for the detection of LTBI, but now in vitro T-cell-based IFN-*γ* assays that are highly specific for mycobacterium tuberculosis (Mtb) have been developed as alternative diagnostic test [3]. In contrast to TST, T-cell assays are unaffected by previous BCG vaccination and infection with most environmental nontuberculous mycobacteria [4]. QuantiFERON-TB Gold in tube (QFT-IT) (Cellestis, Carnegie, Australia) and T-SPOT. TB (Oxford Immunotec, Oxford, UK) are two types of commercially available T-cell assay. QuantiFERON-TB Gold (QFT; second generation) and QFT-IT (third generation) are whole blood assays based on enzyme-linked immunosorbent assay, whereas the T-SPOT. TB uses peripheral blood mononuclear cells for antigen stimulation in an enzyme-linked immunospot assay. Although there is no gold standard for LTBI diagnosis, when surrogate markers of recent exposure to Mtb are used, T-cell assays have been shown in many studies to be as good if not better than the TST in a contact investigation [5, 6]. CDC guidelines recommend the single use of QFT in place of the TST for all circumstances in which the TST is used [7].

The TST is well known to have diminished sensitivity in the setting of immunocompromised hosts. False-negative results in TST were reported, for example, in elderly people and in malnutrition [8, 9]. A recent study suggested that T-cell assays identified more immunocompromised patients with LTBI than did the TST [10]. Because these new T-cell assays rely on measuring specific acquired cell-mediated immune responses, any immunomodulating factors affecting cellular immunity in vivo may impact on the test performance in vitro [11]. However, the correlation between the degree of immunocompetence and specific IFN-*γ* responses is not clear. Furthermore, most studies have analysed data from T-cell assays as dichotomous results using a single cutoff value, and little work has been done on validation of thresholds in consideration of immunological diversity. There is epidemiological evidence to support the use of risk-stratified cutoff values, as in the interpretation of TST [1]. However, no such data exist presently for T-cell assays. We hypothesized that the appropriate positive cutoff value in QFT for diagnosing LTBI would be different in immunocompetent populations and immunosuppressed hosts with impaired cell-mediated immunity. The objective of this study is therefore to identify immune-related clinical indicators associated with the degree of antigen-specific IFN-*γ* production using a large immunologically unselected population with obvious TB infection. Secondly, based on these findings, we revised the threshold values of QFT for populations with different immune status in clinical practice.

## 2. Methods

### 2.1. Participants

From January 2006 to October 2008, new patients and healthy subjects in our hospital verbally consenting to the study were consecutively enrolled after the research protocol was approved by the institutional review board of National Tokyo Hospital (IRB). QFT assay is now approved by national medical insurance in Japan and should be indicated to diagnose TB infection in clinical practice. IRB approves a verbal informed consent for QFT assay. QFT was prescribed by hospital physicians for inpatients and outpatients in any ward, without any influence of the investigators. For all patients recruited into the study, information on their previous medical history, clinical symptoms and signs, and radiological and microbiological data were collected at the time of enrollment. Bronchial lavage fluid samples obtained by bronchofiberscopy were collected and cultured for mycobacteria when it was judged necessary. Clinicians did not take QFT results into account for their final diagnosis. Several routine laboratory tests for clinical diagnosis were simultaneously performed. We also routinely performed QFT on healthy employees at the start of their employment. We collected information on history of prior TB, previous working in any healthcare settings or recent exposure to a patient with active TB and other TB risk factors such as taking immunosuppressive drugs.

### 2.2. Whole Blood QuantiFERON-TB Gold Assay

The test was performed according to the manufacturer's instructions. The interpretation of the results was performed according to the guidelines proposed by CDC [7]. Previously we evaluated that this test would have 74% sensitivity in all patients including HIV-negative immunosuppressive patients [12]. However, it was increased to 88% in patients with peripheral lymphocyte counts ≥1000 cells/mm^3^. Two specialized technicians running the test in our hospital were completely blinded to individual clinical information and final diagnosis.

### 2.3. Statistical Analysis

Associations between antigen-specific IFN-*γ* production in the QFT assay and several clinical characteristics were examined by fitting a linear model. Regression diagnostic exercises included inspection of residual distributions (quantile-quantile and normal-probability plots, residuals-versus-fitted plots), nonlinearity in the functional relationship with predictors (augmented component-versus-residual plots), and examination for collinearity (variance inflation factor). Predictions made from the fitted model were expressed in the original metric with the aid of Duan's smearing estimation. Assay results were subjected to maximum-likelihood logistic regression against the categorized subpopulations. Pairwise differences in logistic regression coefficients were compared at a Type I error rate of 0.05, adjusted for multiple comparisons by the Bonferroni method. Pairwise comparisons were made between areas under the receiver operating characteristic (ROC) curve (AUROC) for the categorized groups. Adjustment for multiple comparisons was made using the Dunn-Sida'k method. *P*  values < .05 were considered significant. Statistical analysis was conducted with Stata, Release 10.1 and 11 (StataCorp LLP, College Station, Texas, U.S.A.), and GraphPad Prism 5 (GraphPad software, San Diego, California).

## 3. Results

### 3.1. Characteristics of the Patients

**Figure 1:**
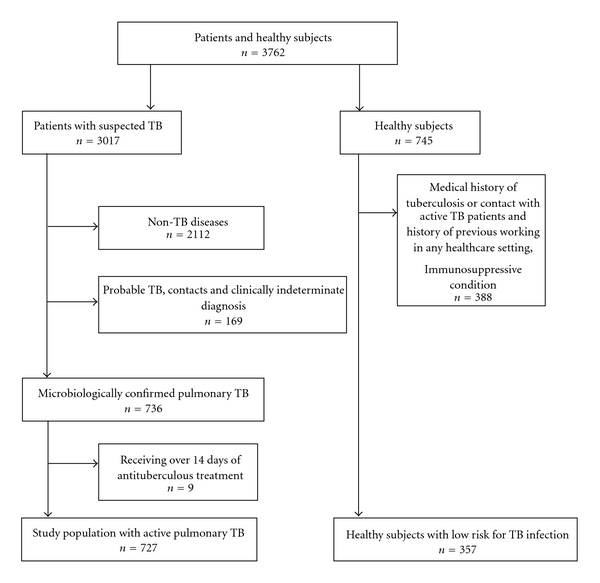
Study participants recruitment profile. TB: tuberculosis.

A total of 3762 subjects underwent initial QFT testing. As shown in Figure 1, of the 3017 TB suspects, 727 patients were finally diagnosed as having definite active pulmonary TB based on the microbiological detection of Mtb from sputum or bronchial lavage fluid obtained using bronchofiberscopy. We excluded patients with probable TB in which no bacteriological evidence was obtained, persons with recent exposure to smear-positive TB patients, and those who had been receiving of antituberculous treatment for more than 14 days at the time of testing. Characteristics of patients with active TB are shown in Table 1. More males than females participated in the study, and most of the study participants were Japanese. The majority (98.9%) was HIV seronegative. Positive sputum smears were found in 552 of 727 patients (75.9%). Clinical immunomodulatory factors pertaining to the TB patients are also shown in Table 1. Patients could have multiple factors at the same time.

### 3.2. Clinical Predictors Related to the Specific IFN-*γ* Response in TB Patients

Associations between specific IFN-*γ* production in unselected TB patients and several patient characteristics were examined by fitting to a linear model. For this, the response variable was the maximum IFN-*γ* after stimulating with ESAT-6 and CFP-10, logarithmically transformed in order to make the distribution of the model residuals approximate more closely to normal and homoscedastic. The following patient characteristics were used as predictors of antigen-stimulated IFN-*γ* production: age, absolute counts of lymphocytes, monocytes, neutrophils and eosinophils, mitogen-stimulated IFN-*γ* production (positive control) and nil (background) negative control IFN-*γ*, and various clinical laboratory test results that might relate to immunocompetence and general condition (serum albumin and blood hemoglobin concentrations), inflammatory status (C-reactive protein level; CRP), serum creatinine concentration, and serum liver enzyme activity levels. None of the predictors were transformed. The values for certain clinical laboratory test results were scaled as needed in order to bring them into similar numerical domains; in practice, this meant that leukogram-related regression coefficients are given in units of thousands of formed elements cells/mm^3^. Three values were missing for albumin, and three for CRP. The total observations available for regression were therefore 721. Regression coefficients, their standard errors, *P* values, and 95% confidence intervals are presented in Table 2. Both background and mitogen-background control conditions are positively associated with antigen-specific IFN-*γ* production (*P* < .0005 and *P* = .006, resp.). Lymphocyte count is likewise positively associated with the test kit response (*P* < .0005). Neutrophil count is negatively associated with specific response (*P* < .0005).

### 3.3. QFT Results in Lymphocyte-Stratified TB Populations

**Figure 2:**
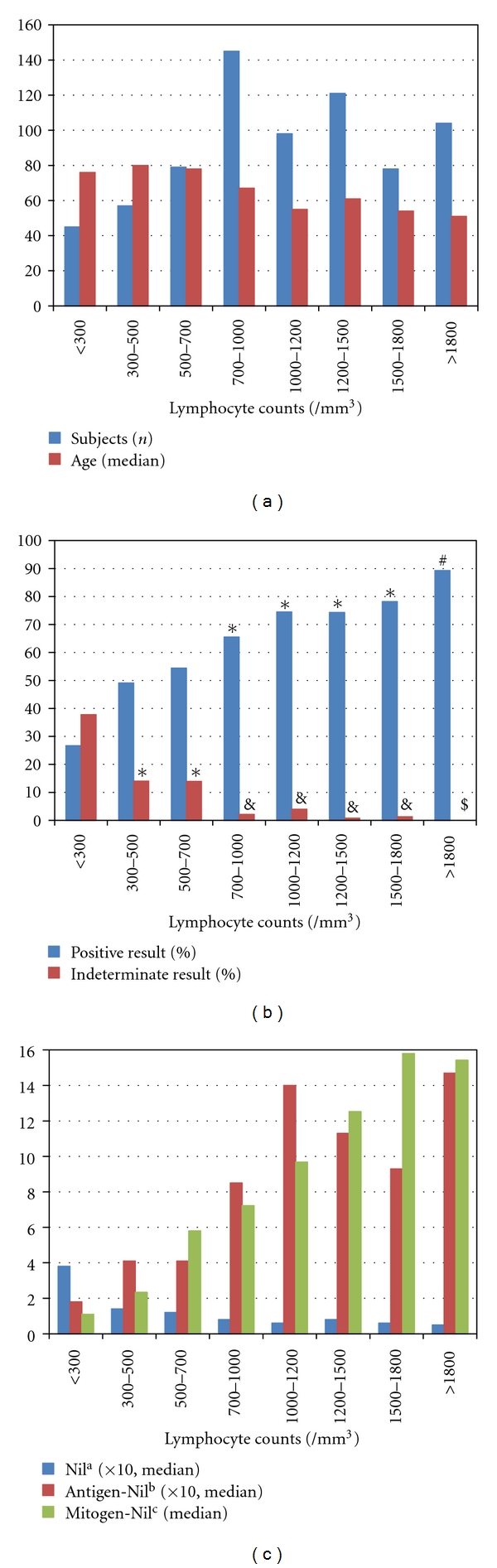
QuantiFERON-TB GOLD results in culture-proven pulmonary tuberculosis subgroups stratified by absolute number of peripheral blood lymphocyte. (a) Numbers and ages of patients divided into eight subgroups based on peripheral lymphocytes. (b) Proportion of QFT results with eight subgroups. *: *P* < .05 versus <300/mm^3^ category. ^&^: *P* < .05 versus <300/mm^3^ category, *P* < .05 versus 300–500/mm^3^ category, and *P* < .05 versus 500–700/mm^3^ category. ^#^: *P* < .05 versus <300/mm^3^ category, *P* < .05 versus 300–500/mm^3^ category, *P* < .05 versus 500–700/mm^3^ category, and *P* < .05 versus 700–1000/mm^3^ category. ^$^: *P* < .05 versus <300/mm^3^ category, *P* < .05 versus 300–500/mm^3^ category, *P* < .05 versus 500–700/mm^3^ category, *P* < .05 versus 700–1000/mm^3^ category, and *P* < .05 versus 1000–1200/mm^3^ category. (c) Antigen-stimulated and mitogen-stimulated IFN-*γ* responses with lymphocyte count (IU/mL). ^a^: Nil (background) IFN-*γ* concentration. ^b^: Difference between the higher IFN-*γ* concentration after stimulation with either antigenic peptides ESAT-6 or CFP-10 and nil (background) IFN-*γ* concentration. ^c^: Difference between the determined IFN-*γ* concentration after stimulation with mitogen and the nil (background) IFN-*γ* concentration.

Next, the TB patient population was divided into eight subgroups based on the absolute number of peripheral blood lymphocyte. Ranges for categories were chosen as 0–299, 300–499, 500–699, 700–999, 1000–1199, 1200–1499, 1500–1799, and >1800 cells/mm^3^ (Figure 2(a)).  Figure 2(b) displays the QFT results for patients according to these eight categories of lymphocyte counts. The proportion of positive assay results (at a cutoff of 0.35 IU/mL) and mitogen-stimulated IFN-*γ* responses were found to be positively associated with lymphocyte count (Figures 2(b) and 2(c)). Conversely, indeterminate assay results showed a negative relationship with lymphocyte count (Figure 2(b)). Indeterminate result rates significantly increased in the categories with less than 700 cells/mm^3^. Most markedly, in severe lymphocytopenia with less than 300 cells/mm^3^, the fraction of test with indeterminate result was 37.8%.

### 3.4. Optimum Thresholds in Lymphocyte-Stratified TB Populations

**Figure 3:**
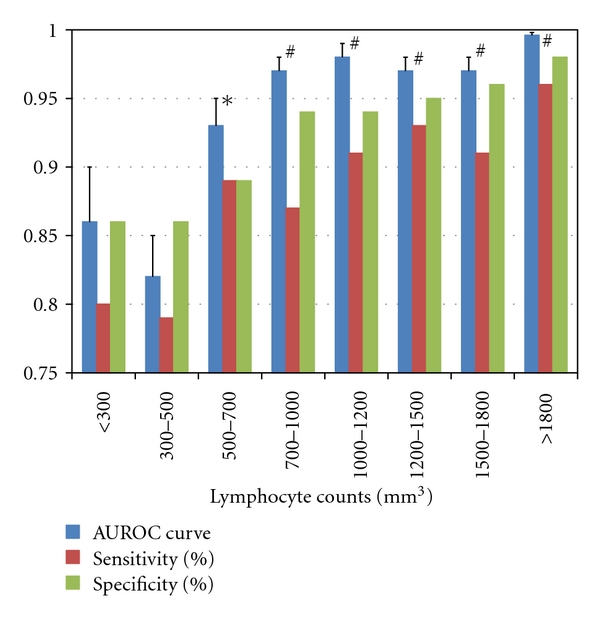
Analysis of area under the receiver operating characteristic curves for lymphocyte-stratified patients groups. AUROC: area under the receiver operating characteristic. Error bar; standard error. *: *P* < .05 versus <300/mm^3^ category and *P* < .05 versus 300–500/mm^3^ category. ^#^: *P* < .05 versus <300/mm^3^ category, *P* < .05 versus 300–500/mm^3^ category, and *P* < .05 versus 500–700/mm^3^ category.

Of the 745 employees in our hospital, 357 immunocompetent individuals had no history of TB disease or contact with active TB patients, and no prior history of working in any health care setting (Figure 1). Median age was 25 years old (range of 21–28). More females (79.3%) than males participated. None of these subjects had any immunosuppressive conditions. All healthy subjects had received BCG vaccination at least once. A positive QFT result was observed in only 3 participants (0.8%), and there were no indeterminate results. Median antigen-specific IFN-*γ* response was 0.01 IU/mL (interquartile range of 0–0.02). Using these QFT data from 357 healthy subjects, for the 8 groups of TB patients stratified by lymphocyte counts as described above, ROC curve analyses were performed to determine the optimum cutoff value (Table 3). In the subgroup with lymphocyte counts of 1800 cells/mm^3^ and over, the AUROC curve was 0.996 and the appropriate cutoff was determined to be 0.19 IU/mL (sensitivity 96% and specificity 98%) (Table 3 and Figure 3). A negative trend of AUROC curve with decreasing lymphocyte counts was observed. Notably, a sudden decrease in AUROC curve was found in patients with lymphocytopenia (less than 700 cells/mm^3^). An appropriate cut-off for moderate lymphocytopenia (700–1500 cells/mm^3^) was determined to be 0.1 IU/mL. Although the test performance was considerably decreased in severe lymphocytopenia group (<700 cells/mm^3^), optimal cutoff value was found to be 0.040 IU/mL.

## 4. Discussion

Different interrelated immunomodulatory factors may generally affect lymphocyte condition in vivo. Malnutrition predisposes to a greater incidence of clinically apparent infection and increased morbidity and mortality due to infection. Depression of circulating lymphocytes and interleukin-2 production following mitogen stimulation has been observed after a fast of only 7 days [13]. Administration of systemic corticosteroid may suppress lymphocyte proliferation and function [14]. T cells from aged animals and humans consistently show depressed responsiveness to mitogens [15]. The presence of a variety of solid tumors is associated with impaired recall delayed-type hypersensitivity and decreased in vitro T-cell proliferation to mitogens [16]. Patients receiving hemodialysis display reduced T-cell function in vitro, diminished antibody production, and compromised neutrophil and dendritic cell function [17]. A recent study has indicated that indeterminate QFT results were increased in HIV-infected individuals with lower CD4^+^ T cell counts [18, 19]. Persons under these clinical immunosuppressive conditions may have a high likelihood of developing active TB disease if they are infected with Mtb. Thus, lymphocytopenia or lymphocyte dysfunction is closely associated with the risk of active TB disease in compromised hosts. The present study revealed that, in an immunologically unselected population with TB infection, absolute lymphocyte counts and mitogen-stimulated IFN-*γ* response are positively associated with the level of antigen-specific IFN-*γ* response in the whole blood T-cell assay. Because CD4^+^ T cells are the major source of IFN-*γ* in T-cell assay, this finding is not unexpected (although our unpublished data using intracellular cytokine-staining method suggested that CD8^+^ T cells also produce IFN-*γ* in some patients). On the other hand, because some patients with normal lymphocyte counts nonetheless had decreased specific IFN-*γ* production, other so far-unidentified lymphocyte count-independent factors such as regulatory T-cell effects [20] could explain decreased IFN-*γ* responses. Although a poor mitogen response was rarely observed in patients with normal lymphocyte counts, good mitogen response could still be mediated in some patients with severe lymphocytopenia. This may suggest that certain leukocytes other than T cells may produce IFN-*γ* in response to whole blood stimulation by mitogen. The present study also suggested that neutrophil count was negatively associated with specific IFN-*γ* response. One possibility could be due to neutrophil-dominant high inflammatory status in advanced tuberculosis with reduced lymphocyte count [21].

Because the risk of LTBI is closely associated with duration of exposure to and proximity to sputum-smear-positive pulmonary TB patient, TB exposure is used as a surrogate for LTBI. However, as there is no proper gold standard for the diagnosis of LTBI, one cannot prove the presence or absence of LTBI [22]. Therefore, for estimating sensitivity and specificity of T-cell assay, untreated patients with culture-positive TB and healthy and low risk individuals without known exposure to TB have generally been used [23, 24].

However, host immunity would be expected to be different between active TB disease and asymptomatic LTBI. Recently, Harari et al. showed that dominant TNF-*α* TB-specific CD4^+^ T-cell response would discriminate between latent infection and active disease [25]. However, they excluded patients with immunosuppression (such as HIV^+^), malnutrition (body weight < 50 kg), and lymphocytepenia (<3000 cells/mm^3^). TB disease severity is associated with greater depression of the total lymphocyte and CD4^+^ T cell counts. The CD4^+^ T-cell counts return to normal levels in most patients after one month of antituberculous chemotherapy [26]. Active TB attenuates antigen-stimulated IFN-*γ* production due to immunosuppression by the disease process itself and migration of specific T cells out of the peripheral blood [27]. This immune suppression in active TB may confound the use of an active TB population as a surrogate for LTBI. We need to select immunocompetent healthy persons known to have TB infection as a surrogate, as far as possible. From the opposite point of view, a population with active TB disease and associated immunosuppression could be used as a surrogate for immunosuppressed hosts with LTBI.

In general, the single cutoff value of 0.35 IU/mL as given in the manufacturer's instruction is uniformly applied to the interpretation of the positive result of QFT at present. Because QFT is used for evaluating individuals suspected of having been infected with TB, it is necessary to decide whether the test result is “positive” or “negative” by using a single cutoff for clinical decision making. However, because the test measures antigen-stimulated IFN-*γ* production from cells in whole blood, the results are inherently continuous variables. Interpretation of the test results including conversions and reversions may require comprehension of nonspecific variability and reproducibility of the continuous data [28]. Moreover, specific IFN-*γ* production might be affected by impaired cell-mediated immunity such as in lymphocytopenia as described above. Here, using the current cutoff value of 0.35 IU/mL, sensitivity was 89% in population with adequate lymphocyte count, whereas in severe (<700 cells/mm^3^) and moderate (700–1500 cells/mm^3^) lymphocytopenia population, sensitivity ranged from 33% to 75% (Table 3). Antigen-specific responses and the test performance were markedly attenuated especially in severe lymphocytopenia. Thus, immunosuppression with severe lymphocytopenia may compromise the test performance. A recent report indicated that QFT sensitivity using the 0.35 IU/mL cutoff in high-incidence settings (69%) might be lower compared with low-burden countries (83%) probably due to several factors including HIV coinfection, advanced disease, and malnutrition [29]. The manufacturer defined assay cut-off 0.35 IU/mL allows for high specificity but likely at some cost in sensitivity, especially under immunosuppressive conditions with lymphocytopenia. Our data suggest that a cut-off of 0.2 IU/mL might be more appropriate for TB-infected immunocompetent subjects with normal lymphocyte count and low inflammatory reaction level which would be consistent with LTBI. Similarly, a cut-off of 0.1 IU/mL maximized the sensitivity without loss of specificity in moderate lymphocytopenic population. In the low burden setting, we propose that the test result should be considered positive if the specific response is greater than 0.1 IU/mL, especially under immunosuppressive conditions associated with lymphocytopenia (Table 4). 

The cutoff value and definition of conversion in the T-cell assays for serial testing is a matter of debate and research both in high- and low-burden settings [30]. Since there is likely to be both individual immunological diversity as well as inherent variability in the determination of IFN-*γ* value, it could be relevant to establish a borderline grey zone [31]. We propose that in immunocompetent healthy subjects, borderline zone on both sides of the optimal cut-off 0.2 IU/mL from our analysis would be relevant (i.e., 0.1–0.3 IU/mL) in low endemic country. Any value less than 0.1 IU/mL is considered “definitely negative”, and any value equal to or greater than 0.3 IU/mL is considered “definitely positive” (Table 4). In Japan, negative criteria for QFT are taken as less than 0.1 IU/mL at present, and the result of the test is considered “equivocal” borderline if the response to the specific antigens is equal or greater than 0.1 and less than 0.35 IU/mL. In the present long-term study, we used the QFT assay with two specific antigens: ESAT-6 and CFP-10. In the QFT-IT assay, new TB-Antigen 7.7 in addition to the two conventional antigens has been introduced and might incrementally improve sensitivity [32, 33]; however, the cut-off 0.35 IU/mL remains unchanged. 

In conclusion, our large longitudinal study revealed that the absolute number of peripheral blood lymphocyte was closely associated with antigen-stimulated IFN-*γ* production in whole blood assay in unselected TB patients. Criteria for assigning a positive result should be stratified to allow for greater performance depending on the immune status of the subjects, especially in immunocompromised patients with impaired cell-mediated immunity who are at increased risk of progression to active TB disease.

## Figures and Tables

**Table 1:** Demographic and baseline clinical characteristics of pulmonary tuberculosis patients.

Total, *n *		727	
Age, median, range		63	15–97
Female, *n*, %		208	28.8
Nationality (other than Japanese), *n*, %		9	1.2
Sputum smear status^a^, *n*, %	0	167	23.0
	1+	187	25.7
	2+	113	15.5
	3+	252	34.7
	unknown	7	1.0
Lung cavitary lesion, *n* (%)		276	38.0
Underlying preexisting conditions^b^, *n*, %			
Administration of immunosuppressant before TB onset^c^		39	5.4
Malignant diseases		44	6.1
Serum albumin less than 3.0 g/dL		248	34.1
Bedridden		78	10.7
Diabetes mellitus		69	9.5
HIV positive		8	1.1
Liver cirrhosis		5	0.7
Renal failure		5	0.7
History of cerebral infarction		17	2.3
Silicosis		3	0.4
Dementia		45	6.2
Alcoholism		15	2.1
Homeless		34	4.7
Drug abuser		0	0.0

Note. ^a^Smear by sputum microscopy: 0 (no acid fast bacilli (AFB) on smear), 1+ (1–99 AFB per 100 field), 2+ (1–10 AFB per field), and 3+ (more than 10 AFB per field).

^b^More than one condition can coexist in the same patient.

^c^Including chronic systemic steroids, antitumor necrosis alpha agents and immunosuppressive agents.

**Table 2:** Regression coefficients and associated statistics.

Predictors	Coefficient	SE	* t*	* P*	95% CI	
					Lower	Upper
Age (y)	−0.0030	0.0027	−1.12	.26	−0.0083	0.0023
background (IU/mL)	0.57	0.089	6.46	<.0005	0.40	0.75
Mitogen background (IU/mL)	0.016	0.0056	2.78	.006	0.0046	0.027
Albumin (g/dL)	0.0055	0.11	0.05	.96	−0.21	0.22
Hemoglobin (g/dL)	−0.023	0.029	−0.79	.43	−0.080	0.034
Neutrophil (1000/mm^3^)	−0.14	0.021	−6.51	<.0005	−0.18	−0.095
Monocyte (1000/mm^3^)	0.26	0.23	1.13	.26	−0.19	0.72
Lymphocyte (1000/mm^3^)	0.61	0.13	4.65	<.0005	0.35	0.87
Eosinophil (1000/mm^3^)	−0.63	0.44	−1.43	.15	−1.5	0.23
CRP (mg/dL)	−0.015	0.012	−1.27	.21	−0.038	0.0081
AST (IU/L)	−0.0023	0.0030	−0.76	.45	−0.0082	0.0036
ALT (IU/L)	0.0047	0.0032	1.48	.14	−0.0015	0.011
Creatinine (mg/dL)	0.18	0.12	1.48	.14	−0.058	0.41
Mitogen × Lymphocyte	−0.0073	0.0034	−2.16	.031	−0.014	−0.00067
Intercept (ln IU/mL)	0.22	0.47				

Note. Regression coefficients for linear regression of log-transformed antigen-stimulated IFN-*γ* production by the QuantiFERON-TB Gold. Wald (*t*) test statistics are based upon 698 degrees of freedom. *P* values are not adjusted for multiplicity. Diagnostic plots do not indicate significant departures from normality for the distribution of residuals or nonlinearity in the predictions. The maximum variance inflation factor (<4) is within an acceptable range. CRP: C-reactive protein; AST: aspartate amino transferase; ALT: alanine aminotransferase; SE: standard error; CI: confidence inter.

**Table 3:** Analysis of area under the receiver operating characteristic curves for lymphocyte-stratified patients groups.

Lymphocyte count/mm^3^	*n *	AUROC curve	95% CI	SE	cutoff value IU/mL	sensitivity %	95% CI	specificity %	95% CI
<300	45	0.86	0.777–0.943	0.04	0.04	80	65.4–90.4	86	81.7–89.2
300–500	57	0.82	0.806–0.938	0.03	0.04	79	66.1–88.6	86	81.7–89.2
500–700	79	0.93^a^	0.883–0.973	0.02	0.05	89	81.0–95.5	89	85.7–92.4
700–1000	145	0.97^b^	0.962–0.987	0.01	0.1	87	80.3–91.9	94	91.2–96.3
1000–1200	98	0.98^b^	0.957–0.994	0.01	0.1	91	83.3–95.7	94	91.2–96.3
1200–1500	121	0.97^b^	0.954–0.993	0.01	0.1	93	87.4–97.1	95	91.8–96.8
1500–1800	78	0.97^b^	0.949–0.989	0.01	0.15	91	82.4–96.3	96	93.2–97.6
>1800	104	0.99^b^	0.992–0.9997	0.002	0.19	96	90.4–98.9	98	96.0–99.2

Note. AUROC: area under the receiver operating characteristic; SE: standard error; CI: Confidence Interval Categories for lymphocyte counts exclude the value shown for the upper end of the range.

^a^: *P* < .05 versus <300/mm^3^ category and *P* < .05 versus 300–500/mm^3^ category.

^b^: *P* < .05 versus <300/mm^3^ category, *P* < .05 versus 300–500/mm^3^ category, and *P* < .05 versus 500–700/mm^3^ category.

**Table 4:** Proposed Immunocompetence-stratified positive criteria and borderline zone for whole blood T-cell assay (QuantiFERON-TB Gold).

Compromised host^a^		Immunocompetent host (adult)		
≥0.1^b^	<0.1	≥0.3	0.1–0.3	<0.1
positive	Negative	postive	borderline	negative

Note: ^a^: compromised hosts with impaired cell-mediated immunity and lymphocytopenia, such as patients receiving chronic immunosuppressive therapy, advanced malignant diseases, malnutrition, chronic renal failure on hemodialysis, HIV infection, and hospitalised elderly. ^b^: IU/ml.

## References

[1] (2000). Targeted tuberculin testing and treatment of latent tuberculosis infection. This official statement of the American Thoracic Society was adopted by the ATS Board of Directors, July 1999. This is a Joint Statement of the American Thoracic Society (ATS) and the Centers for Disease Control and Prevention (CDC). This statement was endorsed by the Council of the Infectious Diseases Society of America. (IDSA), September 1999, and the sections of this statement. *American Journal of Respiratory and Critical Care Medicine*.

[2] Horsburgh CR (2004). Priorities for the treatment of latent tuberculosis infection in the United States. *New England Journal of Medicine*.

[3] Andersen P, Munk ME, Pollock JM, Doherty TM (2000). Specific immune-based diagnosis of tuberculosis. *Lancet*.

[4] Lein AD, von Reyn CF, Ravn P, Horsburgh CR, Alexander LN, Andersen P (1999). Cellular immune responses to ESAT-6 discriminate between patients with pulmonary disease due to Mycobacterium avium complex and those with pulmonary disease due to Mycobacterium tuberculosis. *Clinical and Diagnostic Laboratory Immunology*.

[5] Arend SM, Thijsen SF, Leyten EM (2007). Comparison of two interferon-gamma assays and tuberculin skin test for tracing tuberculosis contacts. *American Journal of Respiratory and Critical Care Medicine*.

[6] Ewer K, Deeks J, Alvarez L (2003). Comparison of T-cell-based assay with tuberculin skin test for diagnosis of Mycobacterium tuberculosis infection in a school tuberculosis outbreak. *Lancet*.

[7] Mazurek GH, Jereb J, Lobue P, Iademarco MF, Metchock B, Vernon A (2005). Guidelines for using the QuantiFERON-TB Gold test for detecting Mycobacterium tuberculosis infection, United States. *Morbidity and Mortality Weekly Report*.

[8] Stead WW, To T (1987). The significance of the tuberculin skin test in elderly persons. *Annals of Internal Medicine*.

[9] Kardjito T, Donosepoetro M, Grange JM (1981). The Mantoux test in tuberculosis: correlations between the diameters of the dermal responses and the serum protein levels. *Tubercle*.

[10] Richeldi L, Losi M, D’Amico R (2009). Performance of tests for latent tuberculosis in different groups of immunocompromised patients. *Chest*.

[11] Ferrara G, Losi M, Meacci M (2005). Routine hospital use of a new commercial whole blood interferon-gamma assay for the diagnosis of tuberculosis infection. *American Journal of Respiratory and Critical Care Medicine*.

[12] Komiya K, Ariga H, Nagai H (2010). Impact of peripheral lymphocyte count on the sensitivity of 2 IFN-gamma release assays, QFT-G and ELISPOT, in patients with pulmonary tuberculosis. *Internal Medicine*.

[13] Sävendahl L, Underwood LE (1997). Decreased interleukin-2 production from cultured peripheral blood mononuclear cells in human acute starvation. *Journal of Clinical Endocrinology and Metabolism*.

[14] Chiang JL, Patterson R, McGillen JJ (1980). Long-term corticosteroid effect on lymphocyte and polymorphonuclear cell function in asthmatics. *Journal of Allergy and Clinical Immunology*.

[15] Schwab R, Walters CA, Weksler ME (1989). Host defense mechanisms and aging. *Seminars in Oncology*.

[16] Kavanaugh DY, Carbone DP (1996). Immunologic dysfunction in cancer. *Hematology/Oncology Clinics of North America*.

[17] de Marie S (1993). Diseases and drug-related interventions affecting host defence. *European Journal of Clinical Microbiology and Infectious Diseases*.

[18] Raby E, Moyo M, Devendra A (2008). The effects of HIV on the sensitivity of a whole blood IFN-gamma release assay in Zambian adults with active tuberculosis. *PLoS ONE*.

[19] Aabye MG, Ravn P, PrayGod G (2009). The impact of HIV infection and CD4 cell count on the performance of an interferon gamma release assay in patients with pulmonary tuberculosis. *PLoS ONE*.

[20] Guyot-Revol V, Innes JA, Hackforth S, Hinks T, Lalvani A (2006). Regulatory T cells are expanded in blood and disease sites in patients with tuberculosis. *American Journal of Respiratory and Critical Care Medicine*.

[21] Berry MP, Graham CM, McNab FW (2010). An interferon-inducible neutrophil-driven blood transcriptional signature in human tuberculosis. *Nature*.

[22] Menzies D, Pai M, Comstock G (2007). Meta-analysis: new tests for the diagnosis of latent tuberculosis infection: areas of uncertainty and recommendations for research. *Annals of Internal Medicine*.

[23] Jeffries DJ, Hill PC, Fox A (2006). Identifying ELISPOT and skin test cut-offs for diagnosis of Mycobacterium tuberculosis infection in the Gambia. *International Journal of Tuberculosis and Lung Disease*.

[24] Mori T, Sakatani M, Yamagishi F (2004). Specific detection of tuberculosis infection: an interferon-gamma-based assay using new antigens. *American Journal of Respiratory and Critical Care Medicine*.

[25] Harari A, Rozot V, Enders FB (2011). Dominant TNF-*α*+ Mycobacterium tuberculosis-specific CD4+ T cell responses discriminate between latent infection and active disease. *Nature Medicine*.

[26] Jones BE, Oo MM, Taikwel EK (1997). CD4 cell counts in human immunodeficiency virus-negative patients with tuberculosis. *Clinical Infectious Diseases*.

[27] Vekemans J, Lienhardt C, Sillah JS (2001). Tuberculosis contacts but not patients have higher gamma interferon responses to ESAT-6 than do community controls in The Gambia. *Infection and Immunity*.

[28] Veerapathran A, Joshi R, Goswami K (2008). T-cell assays for tuberculosis infection: deriving cut-offs for conversions using reproducibility data. *PLoS ONE*.

[29] Dheda K, Smit RZ, Badri M, Pai M (2009). T-cell interferon-gamma release assays for the rapid immunodiagnosis of tuberculosis: clinical utility in high-burden vs. low-burden settings. *Current Opinion in Pulmonary Medicine*.

[30] Pai M, Joshi R, Dogra S (2006). Serial testing of health care workers for tuberculosis using interferon-gamma assay. *American Journal of Respiratory and Critical Care Medicine*.

[31] Pai M, Joshi R, Dogra S (2009). T-cell assay conversions and reversions among household contacts of tuberculosis patients in rural India. *International Journal of Tuberculosis and Lung Disease*.

[32] Harada N, Higuchi K, Yoshiyama T (2008). Comparison of the sensitivity and specificity of two whole blood interferon-gamma assays for M. tuberculosis infection. *Journal of Infection*.

[33] Mahomed H, Hughes EJ, Hawkridge T (2006). Comparison of Mantoux skin test with three generations of a whole blood IFN-gamma assay for tuberculosis infection. *International Journal of Tuberculosis and Lung Disease*.

